# Radial head and neck fractures in children and adolescents

**DOI:** 10.3389/fped.2022.988372

**Published:** 2023-01-20

**Authors:** Miriam Kalbitz, Ina Lackner, Mario Perl, Jochen Pressmar

**Affiliations:** ^1^ Department of Trauma and Orthopedic Surgery, Friedrich-Alexander University Erlangen-Nurnberg, University Hospital Erlangen, Erlangen, Germany; ^2^ Department of Traumatology, Hand-, Plastic- and Reconstructive Surgery, Centre of Surgery, University of Ulm, Ulm, Germany

**Keywords:** children, pediatric fracture, radial head fracture, radial neck fracture, Metaizeau, AO-PCCF

## Abstract

**Background:**

Radial head and neck fractures are a rare entity in pediatric patients. Due to specific characteristics of the blood supply and remodeling potential, the correct diagnosis and initiation of appropriate therapy are crucial for the outcome. Therefore, the aim of this retrospective observational study was to present the outcome of a series of pediatric patients with radial head and neck fractures.

**Methods:**

In total, 67 pediatric and adolescent patients with a fracture of the proximal radius admitted to a Level I Trauma Center (Germany) between 2005 and 2017 were included in this retrospective observational study. Patients were stratified in accordance with the classification of Judet modified by Metaizeau and with the AO Pediatric Comprehensive Classification of Long Bone Fractures (AO-PCCF).

**Results:**

AO-PCCF fracture type of proximal radius was age-dependent. Epiphyseal axis angle and displacement angle correlated significantly. Fractures treated with a K-wire or embrochage centromedullaire elastique stable (ECMES) presented higher displacement angles. The duration of callus formation was dependent on both the reduction technique and fracture displacement. The range of motion after complete fracture consolidation was dependent on the Metaizeau type and reduction technique but independent of the duration of immobilization and physical therapy.

**Conclusion and clinical relevance:**

Both the epiphyseal axis and displacement angle are suitable for measuring the initial fracture displacement in radiographs. Consolidation is dependent on the initial displacement and reduction technique. The mini-open approach leads to a worse reduction result, later callus formation, and a more restricted range of motion in terms of pronation. Furthermore, the range of motion at follow-up is independent of the duration of immobilization and physiotherapy.

## Introduction

Radial head and neck fractures are rare entities in pediatric patients (1, 2). The most common type of lesion of the proximal radius in children is the fracture of the radial neck (3). Characteristic trauma mechanisms are falls on the outstretched arm with supination of the forearm and an associated valgus thrust causing compression on the radio-capitellar joint, followed by falls on the hyperextended elbow and pronation of the forearm, resulting in transmission of kinetic energy through the shaft of the radius and the epiphyseal cartilage, cumulating on the neck of the radius (4). Since much of the radial head in children is cartilaginous, the valgus force on the radius head is transmitted to the weaker physis and metaphysis in the neck region (5, 6). Based on anatomical differences, especially the thick and resilient cartilage of the proximal radial epiphysis in children, the functional outcome in children is worse compared to adults, especially when the radial head intraarticular fracture involves an open physis (Salter–Harris types III and IV) and particularly when intra-articular fractures are initially treated nonoperatively (7). The outstanding correction potential concerning angular deviations in radial head and neck fractures on the one hand and the limited ability to correct lateral misalignment on the other hand in combination with the vulnerability of the vascular supply of the proximal radius make treatment of these fractures challenging. Damaging the vascular supply of the head occurs either in the traumatic event, during reduction (open or closed), or by extensive physiotherapy (8–10). Therefore, the outcome of radial head and neck fractures has been described as satisfactory in only 64% of patients, whereas 5% had fair results and 31% had poor results (11). More recently published studies described good/excellent results in 76% and fair/poor results in 24% (12) and reaching full range of motion after treatment in 71%–73% (13). Periarticular ossification, avascular necrosis, and enlargement of the proximal part of the radius are the most frequently recorded complications (11). More complex fractures with more invasive treatment or inadequate reduction have been linked to higher complication rates and worse outcomes (8, 9, 14). Disturbing the vascular supply by the operative approach and manipulation during reduction may cause stiffness of the elbow, avascular necrosis of the radial head, and growth arrest, which could lead to cubitus valgus, periarticular ossification, or overgrowth of the radial head (15, 16). Thus, correct diagnosis and an appropriate therapeutic approach are essential to avoid long-term consequences like impaired forearm rotation, cubitus valgus, elbow instability, and chronic pain (17). Since 1965, the most commonly used therapy for undisplaced radial head and neck fractures in children is immobilization in a splint without any reduction (3, 6). However, there is an ongoing debate regarding the therapeutic approach to displaced radial neck and head fractures in children (18, 19). In addition, the diagnostic procedures to determine fracture displacement must be reviewed with regard to their accuracy. Accordingly, the displacement of the proximal fragment is determined by using the long axis of the radial shaft as a reference (epiphyseal axis angle) most frequently. However, this approach neglects the individual angulation (20) of the radial neck and the extent of supination of the forearm and further depends on the quality of the x-ray, which, for example, leads to incorrect measurements when interpreting the anterior–posterior image in pronation. Nevertheless, this angle is applied to determine the displacement of the proximal fragment (21). Therefore, the aim of this retrospective observational study was to present the outcome of a series of pediatric patients with radial head and neck fractures. We first hypothesize that there is a positive correlation between the epiphyseal axis angle and displacement angle, indicating that both angles are suitable for measuring the fracture displacement in radiographs of radial head and neck fractures. We further hypothesize that the clinical outcome with regard to the range of motion and consolidation depends on the initial displacement of the fracture, reduction and fixation technique, the duration of cast therapy, and physical therapy. Accordingly, we defined range of motion as primary and callus formation as secondary outcomes of our retrospective study.

## Materials and methods

### Design

We included 67 pediatric and adolescent patients (<17 years) with a radial head and neck fracture admitted to a Level I Trauma Center (Germany) between 2005 and 2017 in this retrospective study. Ethical approval was obtained from the local ethic committee (No. 44/18). Inclusion criteria were age <17 years with a radial head and neck fracture (epiphyseal or metaphyseal) after trauma. Exclusion criteria were defined as age ≥17 years, pathologic fracture, and metabolic/genetic bone disease.

The proximal fractures of the radius were classified based on the fracture location and morphology. Therefore, the AO Pediatric Comprehensive Classification of Long Bone Fractures (AO-PCCF) was utilized (22, 23). This specific pediatric classification code includes the detailed localization of the fracture, the fracture pattern, and the severity (simple/multifragmentary) of the fracture.

Moreover, we stratified the fractures in accordance with the classification of Judet modified by Metaizeau: Grade I no displacement, no angulation; grade II displacement 2/3 of the corpus radii, angulation of <30°; grade III considerable displacement, angulation of 30°–60°; grade IV complete displacement, angulation of 60–90°, and disruption of the ligAmentum anulare (24).

In the case of doubt, x—rays were discussed directly between the radiologist and the surgeon. In general, one preoperative x—ray is followed by an x—ray immediately postoperatively or after casting in the case of conservative therapy. The next follow-up x—ray usually is done 2 weeks later. If required, further x—rays may be taken. Callus formation was defined as the time point when callus formation was clearly visible in the x—ray. Epiphyseal axis angles and displacement angles were measured on initial and follow-up x—rays. Moreover, the time to callus formation was assessed on follow-up x—rays.

We differentiated between epiphyseal and metaphyseal fractures and assessed age distribution, trauma mechanism, treatment strategies, immobilization, and range of motion in the clinical course. The evaluation of the epiphyseal and displacement angles was conducted by an experienced trauma surgeon who is specialized in pediatric fractures.

### Statistics

Data were analyzed by using GraphPad Prism Version 7.0. We performed normality testing with the Shapiro–Wilk test. On normally distributed data, we applied one-way ANOVA followed by Tukey’s multiple comparison test to identify differences between more than two groups. Wilcoxon matched-pairs signed rank tests compared the results of two matched groups. On not normally distributed data, we applied the Kruskal–Wallis test followed by Dunn’s multiple comparison test to identify differences between more than one group and the Mann–Whitney test to compare the results of two groups. For all analyses, *p* < 0.05 was considered statistically significant. Normally distributed data were presented as mean ± standard error of the mean (SEM). Not normally distributed data were presented as median with interquartile range. For correlation analysis, simple linear regression with a 95% confidence interval was performed (Spearman’s test).

## Results

### Epidemiology

The mean age of patients included in this study was 8.5 years (range 1–16 years). Patients with metaphyseal fractures (*n* = 27, 40.3%) were slightly younger (mean 7.9 years, range 1–15 years) than patients with epiphyseal fractures (*n* = 40, 59.7%, mean 8.9 years, range 1–16). There was no statistically significant difference in age between the patients with epiphyseal and metaphyseal fractures (*p* = 0.2084).

**Figure 1:**
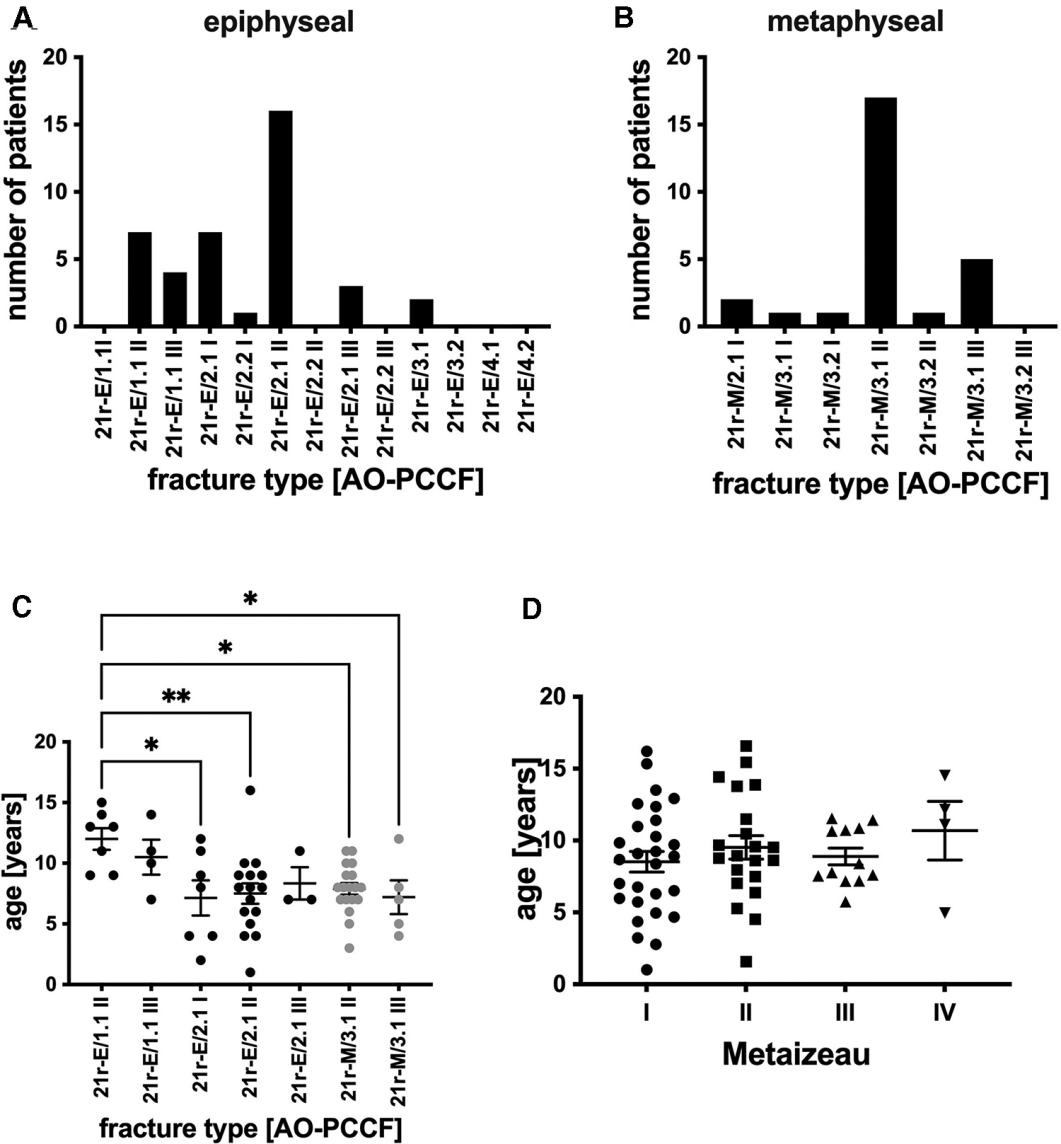
(**A**) Number of patients stratified by AO classification [AO-PCCF] with radial head/epiphyseal radial fractures. (**B**) Number of patients stratified by AO classification [AO-PCCF] with radial neck/metaphyseal radial fractures. The radial head fracture types 21r-E/1.1 I, 21r-E/2.2 II, 21r-E/2.2 III, 21r-E/3.2, 21r-E/4.1, and 21r-E/4.2 were not present as well as the radial neck fracture type 21r-M/3.2 III is therefore not depicted in panels A and B. (**C**) Age in years of patients in respective AO-PCCF fracture types. AO-PCCF fracture types for which two or fewer patients were included are not shown in this panel. (**D**) Age in years in respective Metaizeau types (I–IV). *p < 0.01, **p < 0.001, graphical presentation as mean ± SEM.

In total, 32 (47.8%) of the 67 children were girls and 35 (52.2%) were boys. The right side in 37 (55.2%) and the left side in 30 (44.8%) were fractured. Epiphyseal fractures of the proximal radius occurred most often as Salter/Harris II in 26 patients (65%, 21r-E/2), followed by epiphysiolysis (Salter/Harris I, 21r-E/1.1) in 11 children (27.5%) and Salter/Harris III fracture (21r-E/3) in 2 (5%). Multifragmentary fractures, which are only present in the 21r-E/2 classified fractures of the proximal radius, were rare (2.5%, *n* = 1, 21r-E/2.2) (Figure 1A). The epiphyseal fracture types of the proximal radius 21r-E/1.1 I, 21r-E/2.2 II, 21r-E/2.2 III, 21r-E/3.2, 21r-E/4.1, and 21r-E/4.2 were encountered.

Referring to metaphyseal fractures of the proximal radius (21r-M, Figure 1B), the most common fractures were simple fractures 21r-M/3.1 II (63%, *n* = 17), followed by 21r-M/3.1 III (18.5%, *n* = 5). Torus fractures (21r-M/2.1, 7.4%, *n* = 2) and fractures 21r-M/3.1 I (3.7%, *n* = 1) and 21r-M/3.2 I (3.7%, *n* = 1) were rare as well as multifragmentary fractures 21r-M/3.2 II (3.7%, *n* = 1). 21r-M/3.2 III fractures were not encountered (Figure 1B).

Patients with displaced metaphyseal fractures were significantly younger (21r-M/3.1 II, mean age 7.9 years, and 21r-M/3.1 III, mean age 7.2 years) compared to patients with moderately displaced epiphyseolysis (21r-E/1.1 II, mean age 12 years). Furthermore, patients with nondisplaced epiphyseal Salter/Harris II fractures (21r-E/2.1 I) and 21r-E/2.1 II fractures were significantly younger compared to patients with moderately displaced epiphyseolysis of the proximal radius (21r-E/1.1 II) (Figure 1C). Furthermore, we classified the radial head and neck fractures with regard to the extent of displacement by Metaizeau. We included 29 patients with Metaizeau I, 22 patients with Metaizeau II, 12 patients with Metaizeau III, and 4 patients with Metaizeau IV in the present study. However, there was no statistically significant difference in age when applying the Metaizeau classification (Figure 1D).

### Epiphyseal axis and displacement angle

**Figure 2:**
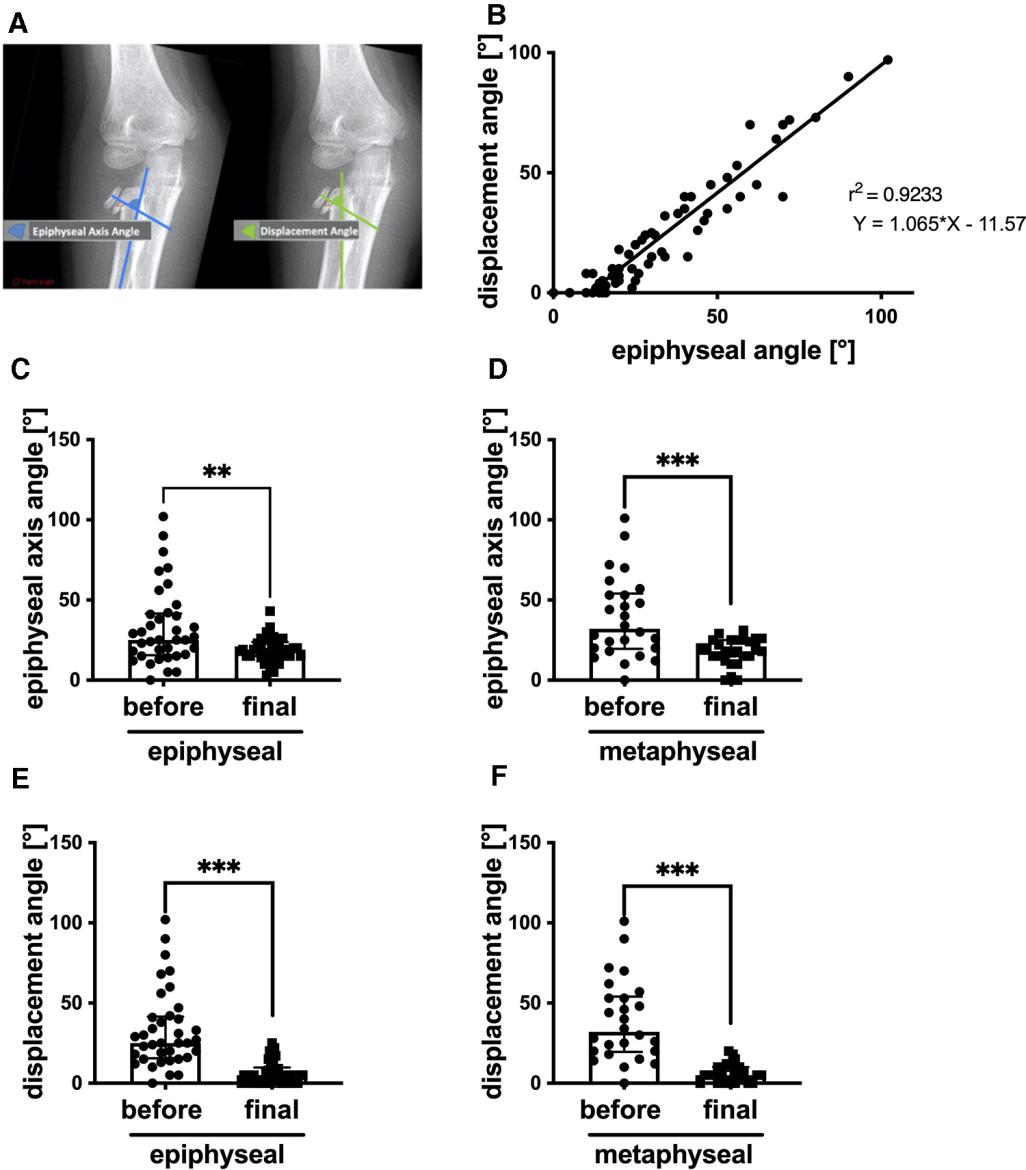
(**A**) Schematic illustration of displacement and epiphyseal axis angle. (**B**) Correlation of displacement and epiphyseal axis angles. (**C**) Epiphyseal axis angle before reduction of epiphyseal/head fracture and final, *n* = 37. (**D**) Epiphyseal axis angle before reduction of metaphyseal/neck fractures and final, *n* = 26. (**E**) Displacement angle before reduction of epiphyseal/head fracture and final, *n* = 37. (**F**) Displacement angle before reduction of metaphyseal/neck fractures and final, *n* = 27. **p < 0.001, ***p < 0.0001, graphical presentation as median with interquartile range. For correlation analysis, simple linear regression with a 95% confidence interval was performed (Spearman test) with *p*-value (two-tailed) < 0.0001.

Since multiple approaches exist to measure the displacement of radial head and neck fractures (25, 26), we first compared a method that is based on the long axis of the radial shaft (epiphyseal axis angle) and the fragment with another method that utilizes the axis of the radial neck as reference for displacement (displacement angle) (Figure 2A). The epiphyseal axis angle and displacement angle were measured on initial x-rays (day 0) and after reduction on follow-up x-rays between day 5 and day 60 (mean 23.12, standard deviation 12.03). We detected a significant correlation between the displacement angle and epiphyseal axis angle (*r*² = 0.92, *p* < 0.0001) before fracture reduction (Figure 2B). The mean epiphyseal axis angle before the reduction of epiphyseal fractures was 33.0°, which was significantly reduced after treatment (*n* = 37, 22.4 ± 2.3 days; mean ± SEM) to a final angle of 18.8° (Figure 2C). Similar observations were made in the case of metaphyseal fractures. The mean angle before reduction was 38.9°, and after treatment, a final epiphyseal axis angle of 17.9° was measured (*n* = 26) (Figure 2D). We focused on the displacement angle, as described above. The mean displacement angle of epiphyseal fractures before reduction was 33°, whereas finally (*n* = 37, 22.4 ± 2.3 days; mean ± SEM), a significantly reduced angle of 6.4° was noted (Figure 2E). The same observation was made after reduction of metaphyseal fractures. The mean displacement angle after final reduction was 6.4° and therefore significantly reduced compared to 38.9° before reduction (*n* = 27) (Figure 2F).

### Treatment

In Table 1, we evaluated the different reduction techniques (no, closed, open, and mini-open) applied in accordance with recommended guidelines oriented on age and displacement angle with regard to indication and their effectiveness in reducing the displacement angle. Forty-two patients were treated conservatively (no operation); 38 of these were without reduction, and 4 patients were with closed reduction and no stabilization. Twenty-five patients were treated in the operation room; 22 of these were with stabilization (K-wire *n* = 11, ECMES *n* = 6, plate *n* = 3, screw *n* = 1, polypin *n* = 1) and the remaining 3 patients were treated with open reduction and no stabilization.

**Table 1:** Displacement angle on initial x-ray, after reduction, and final in regard to treatment as mean ± SEM and time of the final radiograph in days as mean ± SEM.

Observation time point	Reduction	Displacement angle (°) mean ± SEM	Difference in *p* value	Time of the final radiograph (days) mean ± SEM
Primary	No	11.07 ± 1.303		–
	Closed	37.33 ± 7.553	*p* < 0.001^a^	–
	Mini-open reduction	41.25 ± 3.497	*p* < 0.001^a^	–
	Open reduction	60.13 ± 6.504	*p* < 0.00001^a^	–
After reduction	Closed	16.00 ± 4.00		–
	Mini-open reduction	13.00 ± 3.512		–
	Open reduction	5.579 ± 1.243	*p* < 0.001^b^	–
			*p* < 0.0001^d^	
Final	Closed	12.33 ± 3.127	*p* < 0.01^e^	14.67 ± 1.085
	Mini-open reduction	12.25 ± 2.594	*p* < 0.01^f^	39.33 ± 9.871
	Open reduction	4.526 ± 1.143	*p* < 0.0001^d^	22.13 ± 2.257
			*p* < 0.01^c^	

^a^ Significantly different from no reduction within the primary group.

^b^ Significantly different from closed reduction within after reduction group.

^c^ Significantly different from closed reduction within the final group.

^d^ Significantly different from open reduction in the primary group.

^e^ Significantly different from closed reduction in the primary group.

^f^ Significantly different from mini-open reduction in the primary group.

Furthermore, the operative treatment strategies were divided according to the invasive approach into open (*n* = 19), mini-open (*n* = 4), and closed (*n* = 6) reduction. Patients with closed reduction (*n* = 6) were treated with ECMES (*n* = 6) or no stabilization (*n* = 4).

The duration of immobilization in conservatively treated patients ranged from 9 to 29 days (mean 17.5 days). In the case of operation including fracture stabilization, the duration ranged from 11 to 38 days (mean 22.5 days), and in the case of operation without stabilization, the duration ranged from 14 to 28 days (mean 20.2 days).

**Figure 3:**
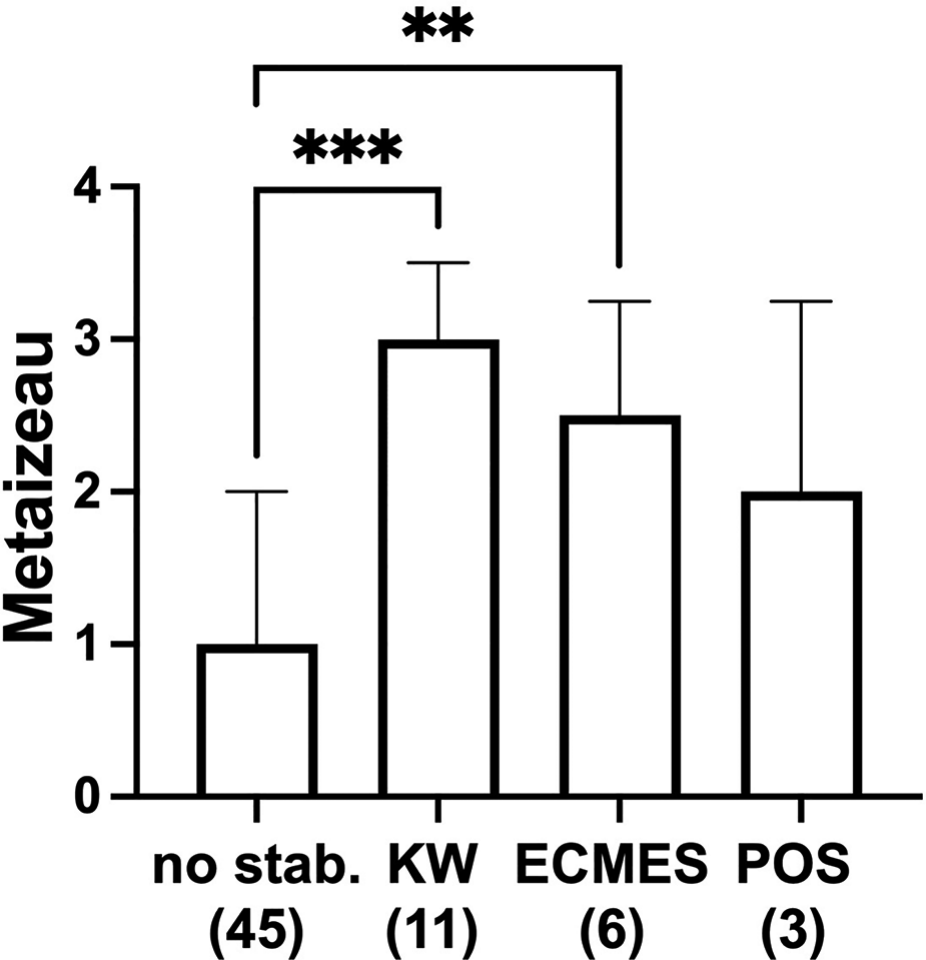
Metaizeau type with regard to treatment strategy no stabilization (no stab. *n* = 45, thereof 38 patients with no reduction, 4 patients with closed reduction, and 3 patients open reduction and no stabilization), KW (K-wire, *n* = 11), ECMES (embrochage centromedullaire elastique stable, *n* = 6), or POS (plate osteosynthesis, *n* = 3). **p < 0.001, ***p < 0.0001, graphical presentation as median ± interquartile range.

As expected, after reduction, all displacement angles were significantly reduced. The final angles were also significantly reduced compared to admission. Closed reduction left higher displacement angles than open reduction, directly after reduction and later at the final observation time point. Fractures with lower displacement angles were treated without reduction. The fractures with high displacement angles were consequently treated with open, closed, or mini-open reduction. The highest displacement angles were treated with open reduction and showed the lowest displacement angles after reduction (Table 1). Fractures treated with ECMES or K-wires had significantly higher displacement angles and therefore a higher Metaizeau classification (II or III) than patients with conservative treatment (Figure 3).

### Callus formation

**Figure 4:**
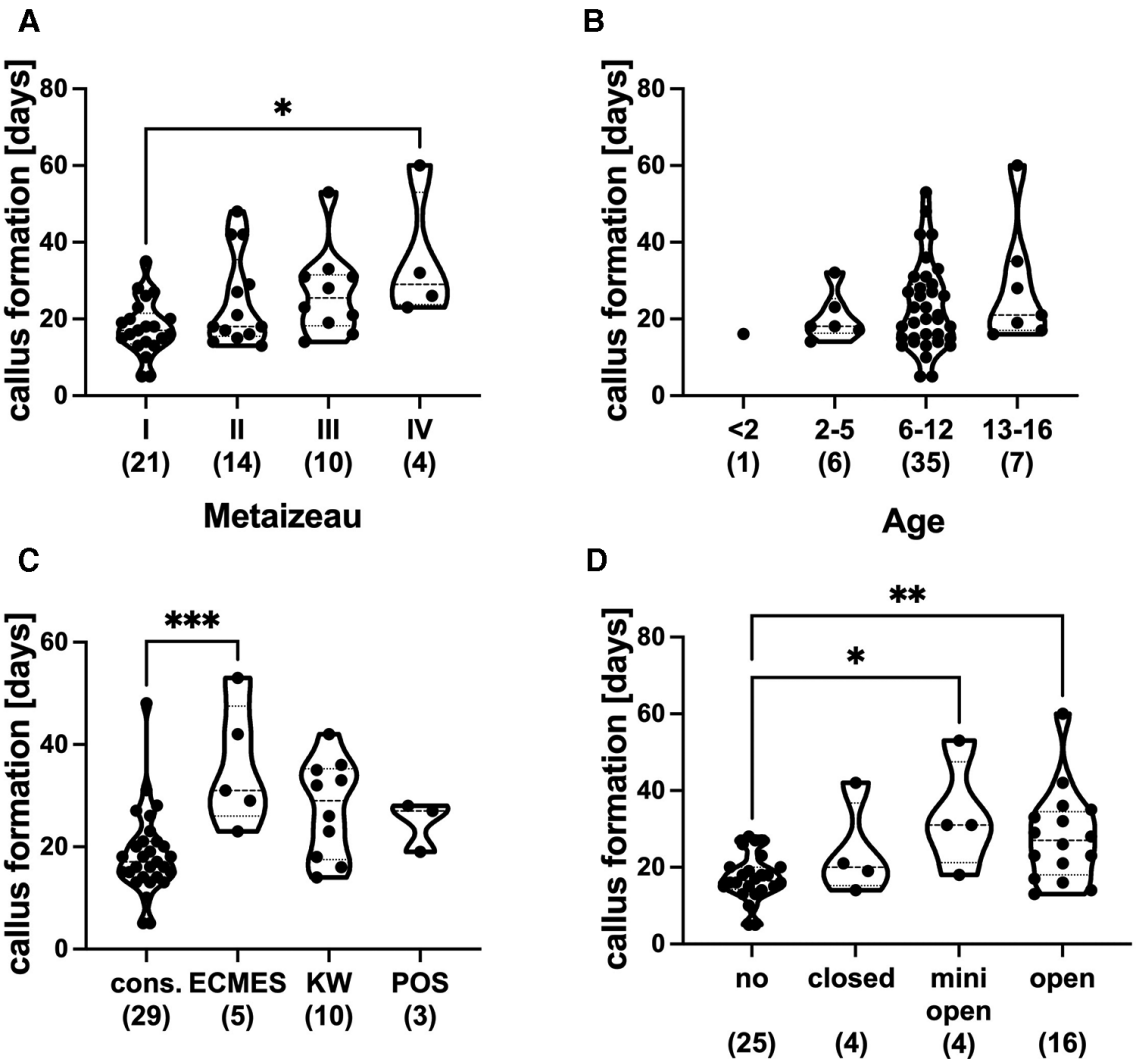
(**A**) Callus formation in days with regard to metaizeau classification (I–IV). (**B**) Callus formation in days depending on age groups in years. (**C**) Callus formation in days with regard to treatment strategy (cons. = conservative treatment and without stabilization, ECMES = embrochage centromedullaire elastique stable, KW = K-wire, POS = plate osteosynthesis). (**D**) Callus formation in days with regard to reduction (no, closed, minimal, open). *p < 0.01, **p < 0.001, graphical presentation as median with interquartile range, ( ) number of patients in respective groups.

Proximal radial fractures classified with Metaizeau IV needed significantly longer time intervals (mean 35 days) for callus formation compared to Metaizeau I fractures (mean 17.3 days) (Figure 4A). The radiographs evaluated to determine callus formation were recorded between day 5 and day 60 (median 20, mean 23.12, 25% percentile 12, 75% percentile 28, standard deviation 12.03). There were no statistically significant differences in callus formation between the age groups (Figure 4B). The duration of callus formation in patients treated with ECMES was significantly longer (mean 35.6 days) cthan conservative treatment (mean 18.5 days) (Figure 4C). More invasive reduction methods (mini-open: mean 33.4 days, open: mean 28.0 days) showed a significantly longer duration of callus formation than fractures treated without reduction (mean 17.3 days) (Figure 4D).

### Range of motion

**Figure 5:**
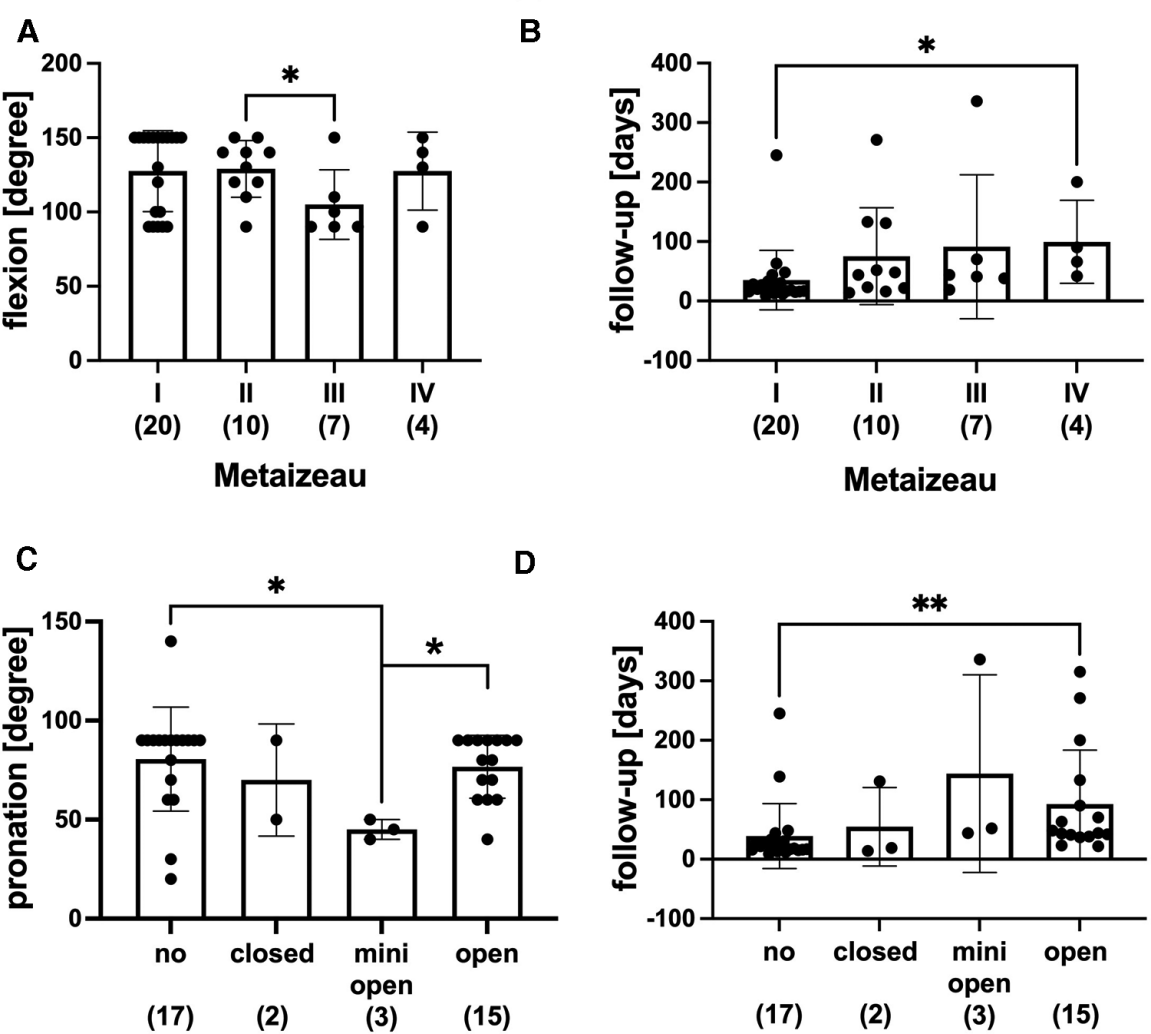
Range of motion after complete fracture consolidation. (**A**) Flexion of the elbow (degree) dependent on Metaizeau type. (**B**) Follow-up duration in days in respective Metaizeau types. (**C**) Pronation of the forearm (degree) depending on the reduction technique (no = without closed, mini = minimal open, open). (**D**) Follow-up duration in days in respective reduction techniques; graphical presentation mean ± SD, *p < 0.01, **p < 0.01, ( ) number of patients in respective groups.

To evaluate the clinical outcome of the different therapeutic strategies, we investigated the extension, flexion, pronation, and supination degree after complete fracture consolidation. Although extension, pronation, and supination did not differ significantly between the Metaizeau fracture types, the flexion was reduced in the Metaizeau type III compared to Metaizeau II (Figure 5A).

**Figure 6:**
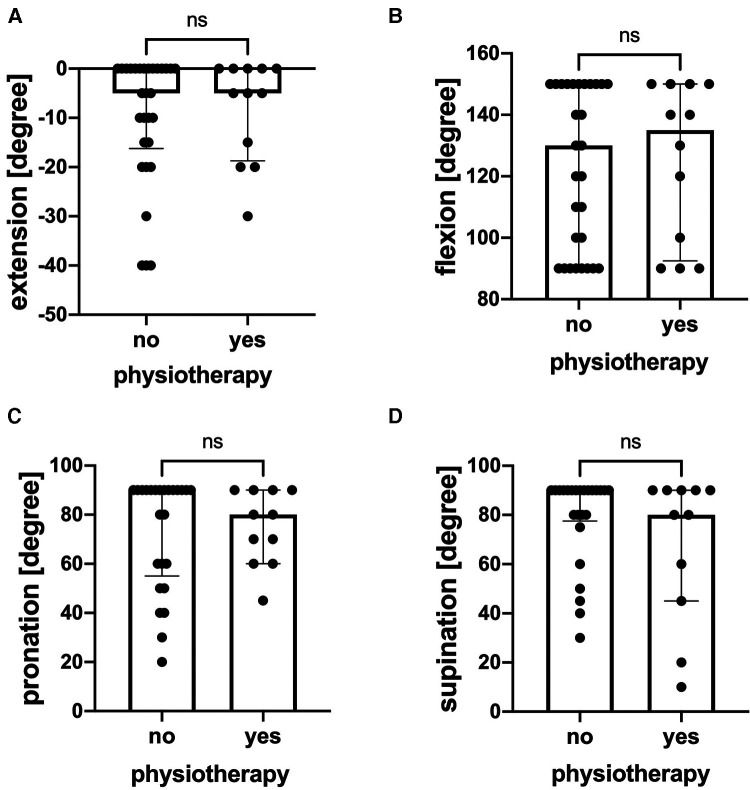
Range of motion after fracture consolidation with and without physiotherapy. (**A**) Extension of the elbow (degree), *n* = 30 without physiotherapy, *n* = 12 with physiotherapy. (**B**) Flexion of the elbow (degree), *n* = 30 without physiotherapy, *n* = 12 with physiotherapy. (**C**) Pronation of the elbow (degree), *n* = 26 without physiotherapy, *n* = 12 with physiotherapy. (**D**) Supination of the elbow (degree), *n* = 26 without physiotherapy, *n* = 12 with physiotherapy. Graphical presentation as median with interquartile range. ns = not significantly different.

Figure 5B depicts the follow-up in different Metaizeau types. There was a statistically significant difference between the follow-up in patients with Metaizeau type IV, which was longer than patients with Metaizeau type I. There was no statistically significant difference between the extension or flexion of the elbow in subgroups of no reduction, closed, mini-open, or open reduction. However, the minimally invasive technique of reduction showed a significantly reduced capability of pronation compared to open reduction and no reduction (Figure 5C). The follow-up duration was significantly longer in patients treated with open reduction than patients with no reduction (Figure 5D). In addition, we analyzed the range of motion dependent on the duration of immobilization and physiotherapy. The range of motion did neither depend on the duration of immobilization nor on the age of the patients (data not shown) or on whether patients received physical therapy or not (Figures 6A–D).

## Discussion

In the present study, we analyzed a large patient collective with proximal fractures of the radius. The AO-PCCF fracture type of proximal radius was age-dependent, and the epiphyseal axis angle and displacement angle correlated significantly. The duration of callus formation was dependent on both the reduction technique and fracture displacement. Furthermore, the range of motion after complete fracture consolidation was dependent on the Metaizeau type and reduction technique but independent of the duration of immobilization and physical therapy.

The mean age of patients with proximal fractures of the radius in the literature is 9–10 years (27, 28) and therefore slightly older than in the present study. Others reported radial head and neck fractures more frequently in girls (28) or boys (26). With regard to fracture type distribution, there were no age differences when applying the Metaizeau classification. However, in the present study, patients with displaced metaphyseal fractures classified by AO-PCCF (21r-M/3.1 II and 21r-M/3.1 III) were significantly younger (7.8 years) than patients with moderately displaced epiphyseolysis (21r-E/1.1 II, 12 years). Furthermore, patients with nondisplaced epiphyseal Salter/Harris II fractures (21rE/2.1 I) were significantly younger than patients with moderately displaced epiphyseolysis of the proximal radius (21r-E/1.1 II). This is, to the best of our knowledge, the first time that significant differences in the age distribution of proximal fractures of the radius were described in detail by utilizing AO-PCCF classification.

To measure displacement in radiographs, most authors described measurements of the angle formed between the displaced fragment and the radial shaft (25, 29). Because multiple approaches exist to measure the displacement of the proximal radial fragment, we compared a method, which is based on the long axis of the radial shaft (epiphyseal axis angle) and the fragment, with another method that utilizes the axis of the radial neck as reference for displacement (displacement angle). Although the epiphyseal axis angle is influenced by individual variations and dependent on the extent of supination and x—ray quality, a significant correlation between epiphyseal axis angle and displacement angle (*r*² = 0.92, *p* < 0.0001) was found before reduction. Furthermore, both the epiphyseal axis angle and the displacement angle were significantly reduced at the completion of treatment in both epiphyseal and metaphyseal fractures. Therefore, in this study, both methods seemed to be appropriate to describe the extent of displacement of the proximal radial fragment in the case of sufficient x-ray quality, which is crucial with respect to the choice of the best therapeutic procedure.

We further showed that the displacement angle was significantly reduced after reduction and at the end of the treatment in all respective groups, closed, minimally invasive, and open reduction. In patients without reduction, final displacement angles were in the same range as those measured in the first radiographs. This is in accordance with the literature (14). Closed reduction of proximal radius fractures in children was mentioned by Kaufmann et al. (1989) (30). Further techniques of closed reduction of fractures of the proximal radius in children were described by others (31, 32). Some authors described closed reduction techniques without stabilization as efficient for displacement angulations up to 60° (26). In our patients, closed reduction without stabilization was successful in all cases (*n* = 4), including one patient with a displacement angle above 60°. A closed reduction yielded satisfactory results within the first 5 days after fracture (33, 34).

Furthermore, the displacement angle was unchanged in patients without reduction at the end of the treatment compared to the first radiograph. Both indicated overall sufficient treatment strategy in our patient collective and in accordance with the literature: Metaizeau suggested that a residual tilt above 10°–15° at the age of 10–12 years or 20°–30° at a younger age could not be remodeled by growth (35).

The treatment of the radial head and neck fractures of the Metaizeau type I and II was described in the literature as predominantly conservative (27). This is in accordance with our results. In the present observation, there was a significant difference between displacement angles in patients treated with closed, minimally invasive, or open reduction. Patients treated with K-wires or ECMES had significantly higher Metaizeau types than those treated conservatively. In accordance with the literature, Metaizeau type IV fractures were frequently treated with open reduction (27), type I fractures according to Metaizeau classification were treated conservatively without reduction, type II–III fractures were treated with reduction, and some of these fractures were trans-fixated by minimally invasive osteosynthesis by Kirschner-wires (K-wires) or ECMES (28). In the present study, open reduction was often necessary in the case of high displacement angles. Displacement angles after reduction and at the end of treatment were significantly smaller compared to closed reduction, which is in contrast to the series of Sessa et al., who described that closed reduction of fracture with closed intramedullary fixation usually provides better angulation results than open reduction (36). In summary, the treatment strategy applied in the present collection is comparable to the approach described in the literature, achieving a satisfactory clinical outcome.

To answer the question of whether the clinical outcome with respect to consolidation and range of motion is dependent on the initial displacement of the fracture, reduction and fixation technique, duration of immobilization, and physical therapy, we first evaluated callus formation in follow-up radiographs. Callus formation occurred later in Metaizeau type IV compared to type I fractures, indicating a correlation between consolidation and initial displacement of the fracture. Furthermore, callus formation occurred significantly later in cases of mini-open and open reduction compared to no reduction and in patients with ECMES treatment compared to the conservative approach. The duration of callus formation in our observation was not significantly longer in older children. As a limitation of this study, we have to mention that due to the retrospective character of the study, x-ray intervals and final follow-up examinations were not standardized. Therefore, the duration of immobilization and callus formation was affected by the out-patient visit appointments, which might lengthen the investigated durations. Furthermore, due to missing or minor quality radiographs in the follow-up x-ray, we had to exclude four patients in assessing the dimension of reduction.

We further evaluated the range of motion dependent on the initial displacement of the fracture, reduction technique, and duration of cast therapy and physical therapy. The range of motion was largely independent of the initial displacement of the fracture. Only flexion was significantly reduced in Metaizau type III compared to type II fractures. In contrast, the range of motion was dependent on the reduction technique. Pronation was significantly reduced in patients with minimally invasive reduction compared to no and open reduction. Furthermore, supination was significantly reduced in all patients with reduction techniques compared to patients with no reduction. Extension and flexion were not dependent on the reduction technique.

Interestingly, the duration of immobilization did not affect the range of motion. Finally, we evaluated the effect of physiotherapy on the range of motion. There was also no effect of the implementation of physiotherapy on the range of motion.

With regard to the outcome of radial head and neck fractures, the age of pediatric patients has been described to affect the results. Older children sustained more severe fractures (14) and had worse outcomes even after stratification for fracture type. Nevertheless, there was no difference in postreduction angles in different fracture types and patient age (14), whereas other studies correlated skeletal maturity with the outcome of radial neck fractures (37–40). In contrast to the literature, in the present report, there was no difference between age groups with regard to the duration of immobilization or callus formation.

## Conclusion

Taken together, we demonstrated that there is a positive correlation between the epiphyseal axis and displacement angle, indicating that both angles are suitable for measuring the initial fracture displacement in radiographs. We further demonstrated that the clinical outcome of epiphyseal and metaphyseal fractures of the proximal radius with regard to consolidation depends on the initial displacement, reduction technique, and fixation. The mini-open approach leads to a worse reduction result, later callus formation, and a more restricted range of motion in terms of pronation. Furthermore, we demonstrated that the range of motion is dependent on the reduction technique and fracture displacement in case of flexion of the elbow but is independent of the duration of immobilization and physiotherapy.

## Data Availability

The original contributions presented in the study are included in the article/Supplementary Material, further inquiries can be directed to the corresponding author/s.

## References

[1] Dormans JP Rang M. Fractures of the olecranon and radial neck in children. Orthop Clin North Am. (1990) 21(2):257–68. 10.1016/S0030-5898(20)31544-3 2326052

[2] Emery KH Zingula SN Anton CG Salisbury SR Tamai J. Pediatric elbow fractures: a new angle on an old topic. Pediatr Radiol. (2016) 46(1):61–6. 10.1007/s00247-015-3439-0 26216157

[3] Henrikson B. Isolated fractures of the proximal end of the radius in children epidemiology, treatment and prognosis. Acta Orthop Scand. (1969) 40(2):246–60. 10.3109/17453676908989505. 5365163

[4] Monson R Black B Reed M. A new closed reduction technique for the treatment of radial neck fractures in children. J Pediatr Orthop. (2009) 29(3):243–7. 10.1097/BPO.0b013e3181990745. 19305273

[5] Vostal O. Radius neck fractures in childhood. Acta Chir Orthop Traumatol Cech. (1970) 37(5):294–302. 5478824

[6] O’Brien PI. Injuries involving the proximal radial epiphysis. Clin Orthop Relat Res. (1965) 41:51–8. 5832738

[7] Leung AG Peterson HA. Fractures of the proximal radial head and neck in children with emphasis on those that involve the articular cartilage. J Pediatr Orthop. (2000) 20(1):7–14 10641680

[8] Vocke AK von Laer LR. Prognosis of proximal radius fractures in the growth period. Unfallchirurg. (1998) 101(4):287–95. 10.1007/s001130050270 9613214

[9] Pesudo JV Aracil J Barcelo M. Leverage method in displaced fractures of the radial neck in children. Clin Orthop Relat Res. (1982) 169:215–8. 10.1097/00003086-198209000-00034 7105583

[10] Hell AK von Laer L. Growth behaviour after fractures of the proximal radius: differences to the rest of the Skeleton. Unfallchirurg. (2014) 117(12):1085–91. 10.1007/s00113-014-2632-1 25427529

[11] Steinberg EL Golomb D Salama R Wientroub S. Radial head and neck fractures in children. J Pediatr Orthop. (1988) 8(1):35–40. 10.1097/01241398-198801000-00009 3335620

[12] Trabelsi A Khalifa MA Brahem R Jedidi M Bouattour K Osman W Radial neck fracture in children: anatomic and functional results of metaizeau technique. Pan Afr Med J. (2020) 36:144. 10.11604/pamj.2020.36.144.22971 32874408 PMC7436640

[13] Badoi A Frech-Dörfler M Häcker FM Mayr J. Influence of immobilization time on functional outcome in radial neck fractures in children. Eur J Pediatr Surg. (2016) 26(6):514–8. 10.1055/s-0035-1566108 26540442

[14] Tan BH Mahadev A. Radial neck fractures in children. J Orthop Surg. (2011) 19(2):209–12. 10.1177/230949901101900216 21857047

[15] Bhargava S Dowell JK Cheah K. A new technique of fixation of displaced proximal radial physeal fracture. Injury. (1999) 30(9):633–6. 10.1016/s0020-1383(99)00160-6 10707234

[16] Jiang H Wu Y Dang Y Qiu Y. Closed reduction using the percutaneous leverage technique and internal fixation with K-wires to treat angulated radial neck fractures in children-case report. Medicine. (2017) 96(1):e5806. 10.1097/md.0000000000005806 28072734 PMC5228694

[17] Ha T Grant S Huntley JS. Monteggia type iv fracture in a child with radial head dislocation irreducible by closed means: a case report. BMC Res Notes. (2014) 7:539. 10.1186/1756-0500-7-539 25129627 PMC4150939

[18] Hemmer J Happiette A Muller F Barbier D Journeau P. Prognostic factors for intramedullary nailing in radial neck fracture in children. Orthop Traumatol Surg Res. (2020) 106(7):1287–91. 10.1016/j.otsr.2020.05.014 32988780

[19] Rouhani A Chavoshi M Sadeghpour A Aslani H Mardani-Kivi M. Outcome of open reduction and Kirschner wire fixation in pediatric radial neck fracture. Clin Shoulder Elb. (2021) 24(4):239–44. 10.5397/cise.2021.00402 34875730 PMC8651599

[20] Nicholson LT Skaggs DL. Proximal radius fractures in children. J Am Acad Orthop Surg. (2019) 27(19):e876–e86. 10.5435/jaaos-d-18-00204 30865025

[21] Macken AA Eygendaal D van Bergen CJ. Diagnosis, treatment and complications of radial head and neck fractures in the pediatric patient. World J Orthop. (2022) 13(3):238–49. 10.5312/wjo.v13.i3.238 35317255 PMC8935328

[22] Slongo TF Audigé L. Fracture and dislocation classification compendium for children: the Ao pediatric comprehensive classification of long bone fractures (Pccf). J Orthop Trauma. (2007) 21(10 Suppl):S135–60. 10.1097/00005131-200711101-00020 18277238

[23] Slongo T Audigé L Lutz N Frick S Schmittenbecher P Hunter J Documentation of fracture severity with the Ao classification of pediatric long-bone fractures. Acta Orthop. (2007) 78(2):247–53. 10.1080/17453670710013753 17464614

[24] Métaizeau JP Prévot J Schmitt M. Reduction and fixation of fractures of the neck of the radious be centro-medullary pinning. Original technic. Rev Chir Orthop Reparatrice Appar Mot. (1980) 66(1):47–9 6447340

[25] Ruf J Craig CL Kuhns L Hall J Farley FA. Radiographic assessment of pediatric proximal radius fractures: interrater and intrarater reliability. J Pediatr Orthop. (2005) 25(5):588–91. 10.1097/01.bpo.0000167082.76212.6d 16199936

[26] Brandão GF Soares CB Teixeira LE Boechat Lde C. Displaced radial neck fractures in children: association of the métaizeau and böhler surgical techniques. J Pediatr Orthop. (2010) 30(2):110–4. 10.1097/BPO.0b013e3181cf118a 20179555

[27] Benz G Roth H. Problem of fracture of the radius head in the child. Z Kinderchir. (1985) 40(5):289–93. 10.1055/s-2008-1059796 4072430

[28] Zeman J Marek O Turek J Seehofnerová A Plánka L. Comparison of two methods of minimally invasive osteosynthesis for proximal radius fractures in paediatric patients. Acta Chir Orthop Traumatol Cech. (2018) 85(4):276–80 30257759

[29] Radomisli TE Rosen AL. Controversies regarding radial neck fractures in children. Clin Orthop Relat Res. (1998) 353:30–9. 10.1097/00003086-199808000-00005 9728157

[30] Kaufman B Rinott MG Tanzman M. Closed reduction of fractures of the proximal radius in children. J Bone Joint Surg. (1989) 71(1):66–7. 10.1302/0301-620x.71b1.2915009 2915009

[31] Augustithis GA Huntley JS. Closed reduction of paediatric radial neck fractures. Ann R Coll Surg Engl. (2015) 97(4):316–7. 10.1308/rcsann.2015.97.4.316 26263944 PMC4473874

[32] Qiao F Jiang F. Closed reduction of severely displaced radial neck fractures in children. BMC Musculoskelet Disord. (2019) 20(1):567. 10.1186/s12891-019-2947-8 31775704 PMC6882240

[33] D’Souza S Vaishya R Klenerman L. Management of radial neck fractures in children: a retrospective analysis of one hundred patients. J Pediatr Orthop. (1993) 13(2):232–8 8459018

[34] Tibone JE Stoltz M. Fractures of the radial head and neck in children. J Bone Joint Surg Am. (1981) 63(1):100–6. 10.2106/00004623-198163010-00013 7451512

[35] Métaizeau JP. Reduction and osteosynthesis of radial neck fractures in children by centromedullary pinning. Injury. (2005) 36 (Suppl 1):A75–7. 10.1016/j.injury.2004.12.016 15652940

[36] Sessa S Lascombes P Prevot J Gagneux E. Fractures of the radial head and associated elbow injuries in children. J Pediatr Orthop B. (1996) 5(3):200–9. 10.1097/01202412-199605030-00012 8866287

[37] Newman JH. Displaced radial neck fractures in children. Injury. (1977) 9(2):114–21. 10.1016/0020-1383(77)90004-3 591044

[38] Gao GX Zhang RY. Radial neck fracture in children. Chin Med J. (1984) 97(12):893–6 6443284

[39] Evans MC Graham HK. Radial neck fractures in children: a management algorithm. J Pediatr Orthop B. (1999) 8(2):93–9 10218168

[40] Steele JA Graham HK. Angulated radial neck fractures in children. A prospective study of percutaneous reduction. J Bone Joint Surg. (1992) 74(5):760–4. 10.1302/0301-620x.74b5.1527130 1527130

